# Development and evaluation of a measure of patient-reported symptoms of Blepharitis

**DOI:** 10.1186/s12955-018-0839-5

**Published:** 2018-01-11

**Authors:** Kamran Hosseini, Linda B. Bourque, Ron D. Hays

**Affiliations:** 1 InSite Vision, Clinical and Regulatory Affairs, 965 Atlantic Avenue, Alameda, CA 94501 USA; 2 UCLA Department of Community Health Sciences, 10833 Le Conte Avenue, 41-230 CHS, Los Angeles, CA 90095-1772 USA; 3 UCLA Department of Medicine, Division of General Internal Medicine & Health Services Research, 911 Broxton Avenue, Los Angeles, CA 90024 USA

**Keywords:** Blepharitis, Symptoms, Reliability, Validity

## Abstract

**Background:**

Blepharitis is an ocular surface disease and chronic ophthalmic condition. This paper reports on the development of psychometric evaluation of a patient-reported measure of blepharitis symptoms.

**Methods:**

Self-reports of 13 blepharitis symptoms collected in a Phase 3 multi-site, randomized, double-masked, 4-arm parallel group, clinical trial of 907 individuals with blepharitis (mean age = 62, range: 19–93; 57% female) were analyzed. Symptoms asked about were: eyes that itch; eyes that burn; eyelids feel heavy or puffy; feel like something is in your eye; dry eyes; gritty eyes; irritated eyes; eyes that tear or water; crusty eyes; flaking from your eyelids; eyelids that are stuck together; red eyes or eyelids; and debris like pieces of skin or dandruff in your eyes.

**Results:**

Categorical factor analyses provided support for two multi-item symptom scales: Irritation (9 items, alpha = 0.88) and Debris (4 items, alpha = 0.85). Spearman-rank order correlations of the Irritation and Debris scales with the Ocular Surface Disease total score were 0.63 and 0.41, respectively (*p*’s < 0.001). Rank-order correlations between ratings of clinicians and self-reports of puffy eyes (*r* = 0.07, *p* < .05), red eyes (*r* = 0.12, *p* < .001), debris (*r* = 0.03, *p* > 0.05), and irritation (*r* = 0.47, *p* < .001).

**Conclusions:**

This study provides support for the psychometric properties and construct validity of the Irritation and Debris scales for assessing symptoms of blepharitis. The associations between the self-reports and clinician ratings of 4 symptoms indicate substantial unique information in the new self-reported symptom items.

**Trial registration:**

The trial was registered at ClinicalTrials.gov under the registry number NCT01408082.

## Background

Blepharitis is an ocular surface disease commonly associated with eyelid inflammation and secondary ocular irritation. The disease is chronic, difficult to manage, recurs often, and its chronicity can lead to scarring of the eyelid and loss of proper eyelid and tear-film function. Self-reported symptoms are important because patients rather than physicians or other health professionals are the best source of this information [1]. Symptoms are often included in measures of health-related quality of life (HRQOL). For example, the widely used SF-36 health survey includes items assessing pain and fatigue. Self-reports of symptoms are particularly useful for ocular surface diseases because conventional clinical assessments are unavailable, technically challenging, expensive or burdensome to the patient.

Self-report measures targeted at ocular surface disease include the McMonnies Dry Eye Index [2, 3], the Ocular Surface Disease Index (OCDI) [4], the Dry Eye Questionnaire (DEQ 2001) [5], the Impact of Dry Eye on Everyday Life (IDEEL) [6], and the Subjective Evaluation of Symptom Dryness (SESoD) item [7]. Each of these measures include questions about ocular symptoms, but none of them fully capture the symptoms experienced by persons diagnosed with active blepharitis.

This paper reports the development and psychometric evaluation of a measure of blepharitis symptoms, the BLepharItIS Symptom (BLISS) measure. The BLISS is designed for use with a large multi-site clinical sample of individuals pre-screened to have blepharitis.

## Methods

InSite Vision Inc. conducted a Phase 3 multi-site, randomized, double-masked, 4-arm, parallel group, clinical trial of 907 subjects with clinically diagnosed blepharitis. All enrolled subjects had used lid-scrub therapy prior to study enrollment and were at least 18 years of age; sex and race were not considered in the inclusion criteria. All subjects had best-corrected visual acuity of 20/100 or better in each eye, intraocular pressure (IOP) of #22 mmHg in either eye, had used lid scrubs for at least 1 week prior to the day 1 study visit, and had a clinical diagnosis of blepharitis.

Among the study-exclusion criteria were previous eyelid surgery within 12 months of study entry that would interfere with study parameters, as determined by the investigator of record; acute ocular infection (bacterial, viral, or fungal) or active ocular inflammation other than blepharitis in the study eye; used topical corticosteroid or topical ophthalmic medications within 14 days before study enrollment; used any non-diagnostic topical ophthalmic solution in the study eye within 2 h before study enrollment; used eye makeup during the dosing period; had any clinically significant lash or lid abnormality other than blepharitis in the study eye; or had moderate-to-severe dry eye in the study eye.

The study questionnaire was available in English and Spanish. Assigned office and research staff provided the questionnaire to study participants during their first study visit. Participants self-administered the questionnaire throughout the study in a private room at the investigational site. Investigators were instructed to not be present during the administration of the questionnaire. There were no case reports from any of the investigational sites citing any issues with a non- or limited English speaking patients. Subjects completed the BLISS at screening, 7 days later at the beginning of treatment, and 15 days later at the end of treatment. This paper focuses on data collected at the beginning and end of treatment. Treatment group comparisons are reported elsewhere [8].

The study was approved by the Copernicus Group IRB.

### Sample and clinical measures

At each visit, investigators at the clinical sites assessed eyelid redness, eyelid swelling, and eyelid debris using the following 0–3 (four categories) rating scales.

- Eyelid redness: 0, None = normal age-related lid coloration; 1, Mild = pink capillary involvement along the lid edge, no patches of confluent capillary redness throughout the lid edge; 2, Moderate = deep pink or red confluent capillary redness present locally along the lid edge; or 3, Severe = deep red, diffuse confluent capillary redness present along the lid edge.
- Eyelid swelling: 0 = None; 1 = Mild; 2 = Moderate; or 3 = Severe.
- Eyelid debris: 0 = no lid/lash debris; 1 = < $$ \raisebox{1ex}{$1$}\!\left/ \!\raisebox{-1ex}{$3$}\right. $$ of lid/lash has collarettes or debris; 2 = > $$ \raisebox{1ex}{$1$}\!\left/ \!\raisebox{-1ex}{$3$}\right. $$ of lid/lash has collarettes or debris; or 3 = $$ \raisebox{1ex}{$2$}\!\left/ \!\raisebox{-1ex}{$3$}\right. $$ of lid lash has collarettes or debris.
- Eyelid irritation: Clinicians asked subjects to grade their present eyelid irritation according to the following scale: 0 = almost none of the time - ≤ 25% of the time; 1 = occasionally – 26-50% of the time; 2 = frequently – 51-75% of the time; or 3 = almost all of the time - ≥76% of the time.

To qualify for the study, subjects at enrollment had to have a combined score of 5 or more in at least one eye, and that eye had to have a score of 1 or greater for eyelid redness and for eyelid irritation. Subjects were required to use standard lid scrubs for 1 week and if their clinical blepharitis score remained ≥5, they were admitted to the study and randomized to treatment. Subjects were screened between November 9, 2011, and September 5, 2012. Data reported here are for the 907 subjects who completed questionnaires at screening, and at the beginning and end of treatment.

### Questionnaire

The BLISS had 13 items assessing blepharitis symptoms developed through review of available clinical reports, other research, and consultation with health care providers active in the diagnosis and treatment of patients with blepharitis. The items were evaluated for comprehensiveness iteratively by investigators at Insight Vision. The items were also reviewed by representatives of the Food and Drug Administration (FDA) Division of Transplant and Ophthalmology Products. For example, the FDA expressed a preference for assessing patient-reported symptom questions using a very recent recall interval (“today”) during the study team’s preliminary discussions with them. Hence, the 13 items asked patients to:

Please think about your eyes today. Are you having any of the following problems with your eyes today? Would you say none of the time, occasionally, frequently, or all of the time?

Symptoms asked about were: eyes that itch; eyes that burn; eyelids feel heavy or puffy; feel like something is in your eye; dry eyes; gritty eyes; irritated eyes; eyes that tear or water; crusty eyes; flaking from your eyelids; eyelids that are stuck together; red eyes or eyelids; and debris like pieces of skin or dandruff in your eyes.

We also administered the 12-item Ocular Surface Disease Index (OSDI) [4]: Have you experienced any of the following today.. . eyes that are sensitive to light; eyes that feel gritty; painful or sore eyes; blurred vision; or poor vision? Are problems with your eyes limiting you in performing any of the following today. .. reading; driving at night; working with a computer or bank machine (ATM); and watching TV? Do your eyes feel uncomfortable in any of the following situations today. .. windy conditions; places or areas with low humidity (very dry); areas that are air conditioned?

Answer responses provided *are: all of the time; most of the time; half of the time; some of the time; none of the time; or not applicable.* Scores for the 12-item OSDI were created using standard scoring recommendations: http://www.dryeyezone.com/documents/osdi.pdf. Internal consistency reliability (Cronbach’s alpha) for the 12-item OSDI scale (*n* = 571) was 0.91.

We also administered the widely used single item rating of general health recommended by the Institute of Medicine’s Committee on the State of the USA Health Indicators to assess global physical and mental health [9]: In general, would you say your health is excellent, very good, good, fair or poor?

We hypothesized that the BLISS would be strongly associated with the OSDI and less strongly related to the general health item.

### Analysis plan

Frequencies for the 13 symptoms items and test-retest reliability (Spearman rank-order correlations) from screening to 7 days later at the beginning of treatment were estimated. Exploratory factor analysis was conducted for the symptom items with principal axis extraction and PROMAX rotation, and confirmed with categorical confirmatory factor analysis using Mplus, Version 7. Spearman correlations were computed between Irritation and Debris symptom scales with the OSDI total score and the general health rating item.

To evaluate the uniqueness of information provided by the BLISS items, we compared patient responses to questions about irritated eyes, heavy puffy eyelids, red eyelids and debris in the eyes to clinical assessments of irritation, swelling, red eyelids, and debris.

## Results

The mean age of the sample is 62 with a range of 19 to 93 years. The median completed education was some post high school training, and 57% of the sample was female.

### Descriptive statistics

Frequencies and test-retest correlations for each of the 13 symptoms are reported in Table 1. With a possible response range of 0 *(none of the time*) to 3 (*all of the time*), modes and medians of 1 or 2 were observed. The most frequently reported symptoms were irritated eyes and red eyes or eyelids.

**Table 1:** Frequencies for 13 Blepharitis Symptoms at Beginning of Treatment and Test-Retest Reliability Over 7 Days (Screening Untill Beginning of Treatment)

Symptoms^a^	None of the time	Occasionally	Frequently	All of the time	Spearman correlation^1^
Eyes that itch	14%	38%	36%	12%	0.58
Eyes that burn	25%	34%	30%	10%	0.62
Eyelids feel heavy or puffy	27%	30%	26%	17%	0.66
Feel like something in your eye	20%	36%	31%	13%	0.61
Dry eyes	24%	26%	31%	19%	0.74
Gritty eyes	27%	32%	28%	12%	0.65
Irritated eyes	15%	30%	35%	19%	0.60
Eyes that tear or water	27%	33%	27%	13%	0.69
Crusty eyes	34%	36%	23%	8%	0.61
Flaking from your eyelids	49%	27%	18%	6%	0.68
Eyelids that are stuck together	60%	27%	11%	3%	0.64
Red eyes or eyelids	23%	26%	29%	23%	0.71
Debris like pieces of skin or dandruff in your eyes	50%	27%	16%	7%	0.64

^a^Number of responses to symptoms items ranged from 897 to 904

^1^All correlations significant at *p* < .001

### Test-retest reliability

Spearman rank-order correlations between screening and the beginning of treatment ranged from 0.58 for eyes that itch to 0.74 for dry eyes (Table 1).

### Factor analysis

The first eigenvalues for the first two factors exceeded one (5.6 and 1.3). The Promax rotated two-factor solution suggested a: 1) 9-item factor (Irritation) comprised of: eyes that itch, eyes that burn, eyelids feel heavy or puffy, feel like something in eye, dry eyes, irritated eyes, gritty eyes, red eyes or eyelids, and eyes that tear or water; and 2) a 4-item factor (debris) comprised of crusty eyes, flaking from eyelids, eyelids that are stuck together, and debris like pieces of skin or dandruff in your eyes.

Categorical confirmatory factor analysis at screening provided support for the eye irritation and eyelid debris factors (Table 2). The two-factor model represented a significant improvement in fit over a single factor model (chi square difference of 10,043 with 12 degrees of freedom). The two-factor model fit the data well: the Comparative Fit Index was 0.954 and the Root Mean Square Error of Approximation was 0.092. The estimated correlation between the two factors was 0.72.

**Table 2:** Categorical Confirmatory Factor Analysis at Screening (*n* = 907)

Symptoms	Factor 1		Factor 2		R Squared
	Estimate	Standard Error	Estimate	Standard Error	
Eyes that itch	0.72	0.019			0.52
Eyes that burn	0.78	0.015			0.61
Eyelids feel heavy or puffy	0.69	0.020			0.47
Feel like something is in your eye	0.76	0.016			0.58
Dry eyes	0.65	0.021			0.42
Gritty eyes	0.80	0.014			0.64
Irritated eyes	0.83	0.012			0.69
Eyes that tear or water	0.57	0.024			0.32
Red eyes or eyelids	0.61	0.023			0.38
Crusty eyes			0.83	0.016	0.69
Flaking from your eyelids			0.79	0.018	0.62
Eyelids that are stuck together			0.73	0.026	0.53
Debris like pieces of skin or dandruff in your eyes			0.82	0.019	0.67

Note: χ^2^ (64 dfs) = 551. Comparative fit index = 0.954; Root mean square error of approximation = 0.092 with 90% confidence interval of 0.085 to 0.099). Estimated correlation between two factors was 0.72

Scores were calculated by averaging responses to items in each scale for the 896 subjects who answered all of the questions for the Irritation scale and the 900 subjects who answered all of the questions for the Debris scale at the beginning of treatment. The mean scale score for Irritation was 1.38 (SD = 0.71) and for Debris was 0.81 (SD = 0.75). Cronbach’s alpha was 0.88 for Irritation and 0.85 for Debris. Spearman rank-order correlations between these scales and the OSDI were 0.63 and 0.41 for Irritation and Depris, respectively (p’s < 0.001). Rank-order correlations with the general health rating item were not statistically significant (rho = 0.01 for Irritation and rho = 0.00 for Debris).

### Associations of BLISS items with clinician ratings

As noted above, investigators assessed eyelid redness, eyelid swelling, and eyelid debris, and asked subjects to grade their eyelid irritation at every visit using 0–3 response scales. Four of the questions in the BLISS asked analogous questions about red eyes or eyelids, eyelids feel heavy or puffy, debris like pieces of skin or dandruff in your eyes, and irritated eyes using similar 0–3 (four point) rating scales. Spearman rank-order correlations between clinical ratings and BLISS scores for puffy eyes (*r* = 0.07, *p* < .05), and red eyes (*r* = 0.12, *p* < .001) were small. The correlation between the clinical rating and the question about debris was *r* = 0.03 (p = NS). Only the correlation between the question on irritation and the clinical score on irritation (*r* = 0.47, *p* < .001) was large (i.e., > = 0.37).

## Discussion

Thirteen questions were developed to measure the symptoms experienced by persons with blepharitis. The test-retest reliability for individual items ranged from 0.59 to 0.74, which is within the .40 to .74 reported by Hahn et al. [1] to represent moderate to good reliability (p. 1247).

Two distinct but correlated multi-item scales were created from the 13 items: 1) Irritation, a 9-item scale consisting of itching, burning, puffy eyelids, irritated eyes, red eyes) characteristic of a mild inflammation; 2) Debris, a 4-item scale consisting of crusty eyes, flaking from eyelids, eyelids stuck, and debris. Only 2% of responses for Irritation were at the lowest value of 0 and 1% at the highest value of 3; 24% of scores on Debris were at 0 and 1% at 3.

The associations of the four clinical ratings of blepharitis signs and symptoms with the four analogous self-report items were small. The highest correlation was between the clinical measure and the patient report of irritation. Unlike the other three clinical measures, subjects were asked a question by the clinician about their irritation. It is instructive to note that the highest correlations for four of the other eight questions in the Irritation scale (eyes that burn; eyelids feel heavy or puffy; dry eyes; red eyes or eyelids) were with the question about irritation rather than with any of the other questions; they ranged from 0.52 to 0.65.

The disconnect between the four questions and the four clinical measures used in this study is consistent with studies of other conditions such as dry eye [10]. Hahn et al. [1] reviewed the associations between patient self-reports and biological, physiological, and physician assessments of health status, and found that there were substantial discrepancies.

Substantial correlations were observed between the two blepharitis scales and the OSDI total scale. This suggests that persons with active blepharitis symptoms were more likely to have problems reading, driving at night, working with a computer or bank machine (ATM), and watching television, and that windy conditions, low humidity, and air conditioning tend to exacerbate blepharitis symptoms. The blepharitis scales were uncorrelated with ratings of general health.

The BLISS can be used in future studies to evaluate symptoms in patients with blepharitis. However, future work is needed to evaluate whether a longer recall interval (other than “today”) leads to richer information about the impact of blepharitis.

### Limitations

The sample used for this study represents both its greatest strength and its greatest limitation. The 907 participants in this clinical trial were screened by clinicians with a standardized protocol and determined to have blepharitis. As such it represents one of the largest samples reported in the ophthalmic literature to have been screened to have blepharitis. In spite of this, an average of 12% (range: 3%–23%) of respondents reported symptoms at the highest level on the 13 questions at the beginning of treatment. This meant that distributions were good for developing scales, but suggested that either symptoms of blepharitis are not as disabling as sometimes assumed or that the restriction to asking only about symptoms experienced today affected distributions.

Potential subjects were screened to exclude those with an acute ocular infection, an active ocular inflammation other than blepharitis, clinically significant lash or lid abnormalities other than blepharitis, and severe dry eye. Despite this, 19% of respondents said they experienced dry eye “all of the time” at the beginning of treatment, and between 8% and 15% said they experienced the five symptoms included in the OSDI all of the time. Shiffman et al. [4] screened 109 subjects for dry eye symptoms. Yet, 44% of the subjects were found to have concomitant ocular conditions including blepharitis and meibomian gland dysfunction. Similar comorbidities may exist in this sample.

A final study limitation is the timing of qualitative data collection. After the trial, four focus groups with a total of 30 individuals with blepharitis were conducted. In addition, 10 one-on-one cognitive interviews were performed. Input from the focus groups and cognitive interview participants confirmed that the questions covered the range of symptoms that persons with blepharitis experience. That is, the 12 symptoms were deemed important and no additional symptoms of blepharitis were suggested by participants upon prompting.

## Conclusions

Thirteen questions about symptoms associated with blepharitis were designed. Focus groups and cognitive interviews conducted after the study indicated that the questions covered the range of symptoms that persons with blepharitis experience. Two scales (Irritation and Debris) were developed that demonstrated good psychometric characteristics. Associations between four of the questions and clinical measures included in the study were low. Associations between the Irritation and Debris blepharitis scales and self-rated general health were also low and may reflect the fact that the retrospective window used for the new items was limited to a single day. In contrast, the Irritation and Debris scales were highly correlated with the 12-item OSDI.

The extent to which these findings can be replicated in other similarly-screened samples should be examined. In addition, we recommend that the two blepharitis scales be included in epidemiologic studies or population-based samples to find out whether they identify persons with blepharitis and differentiate them from those without blepharitis. Such studies might also be able to identify the threshold at which disease occurs, and examine the sensitivity and specificity of the measures. Finally, future research should determine whether the two blepharitis scales can be used to differentiate persons with blepharitis from persons with other ocular surface conditions. To do this it may be necessary to group potential participants into disease categories using physiological measures in addition to the kinds of clinician observations used here.

The BLISS is being administered in a Phase III blepharitis study. Results from this study will provide information on the potential utility of this instrument in the clinical settings.
